# Assessment of Oral Health Status in a Prison Population in Northern Portugal

**DOI:** 10.4317/jced.60551

**Published:** 2023-11-01

**Authors:** Mariana Soares, Maria Gonçalves, Paulo Rompante, Filomena Salazar, Luís Monteiro, José-Júlio Pacheco, Marta Relvas

**Affiliations:** 1 UNIPRO, Oral Pathology and Rehabilitation Research Unit, University Institute of Health Sciences (IUCS-CESPU), 4585-116 Gandra, Portugal; 2 Toxicology Research Unit (TOXRUN), (IUCS-CESPU), 4585-116 Gandra, Portugal

## Abstract

**Background:**

Prisoners constitute one of the disadvantaged groups and it is observed that these individuals suffer from poor oral hygiene and the prevalence of oral diseases is higher compared to the general population.

**Material and Methods:**

The aim of this study is to assess the state of the oral health in a prison population in Northern Portugal. A cross-sectional observational study was conducted involving 103 male prisoners with age between 25 and 75 years old. A questionnaire was presented to all the prisoners and an intraoral clinical examination was performed in each of them.

**Results:**

The sample mean age was 41.58 ± 8.94 years. Most participants consume sugary foods, with 32% consuming then on a daily basis. It was noticed that 13.6% of the participants do not brush their teeth. Most of the prisoners smoke (78.6%) and 70 smoke more than 10 cigarettes per day. The mean DMFT was 17.17 ± 8.23 and the component with the highest weight was the number of missing teeth, with a mean value of 13.14 ± 8.32. It was observed that 7 individuals are edentulous and 64 (62.1%) have caries lesions. It has been noted that of the 64 individuals presenting caries lesions, 47 (73.4%) eat sugary foods, however this relationship is not statistically significant. It was found that the prevalence of periodontal health was 26%, gingivitis was 32.3% and periodontitis was 41.7%. Plaque Index was evaluated according to the periodontal condition, it was observed a significantly lower level of plaque index in the periodontally healthy subjects compared to the subjects with gingivitis and the subjects with periodontitis.

**Conclusions:**

The prevalence of oral diseases in this prison population is high, as is the loss of teeth. Dental caries is the most observed oral disease, and periodontitis the most common of the periodontal diseases.

** Key words:**Prisoners, Oral health, Caries, DMFT, Periodontitis, Oral diseases.

## Introduction

Oral health is essential for overall health, well-being, and quality of life. A healthy mouth is the fundamental condition for people to be able to eat, speak and socialize without pain, discomfort, or embarrassment (1).

Despite major advances in oral health worldwide, problems remain in many communities, particularly among the most disadvantaged and vulnerable groups (2).

Prisoners are one of the disadvantaged and vulnerable groups as they are deprived of their liberty (2). The prison population is made up of a high percentage of individuals belonging to marginalized groups, unemployed or people with a low level of education coming from unfavorable and socially excluded environments (2,3). In these groups, as a rule, there is a greater tendency to develop risk behaviors and to assume negligent attitudes towards health. The lack of preventive care, associated with factors such as an inadequate diet and malnutrition, facial trauma due to exposure to violence and excessive consumption of alcohol, tobacco, and illicit substances, leads them to negligence of their oral health (4,5).

Among the existing studies, it has been shown that dental caries and periodontal diseases are the most prevalent oral manifestations in prison settings (6,7). It has been observed that inmates lack oral hygiene status compared to the general population (8). Also, inmates, according to Walsh *et al*. (2008) (9) have more decayed, lost and filled teeth than the general population.

The main objective of the present study is to assess the oral status in a population of prisoners in Northern Portugal, according to 3 specific objectives:

- Determination of the number of decayed, missing and filled permanent teeth (DMFT), as well as the prevalence of dental caries;

- Analyze the prevalence, extension and severity of periodontal disease, according to the 2017 Chicago Workshop;

- Assess potential indicators of the risk of periodontal disease and caries lesions in this population.

## Material and Methods

-Design and Ethical Approval

The present study was conducted the prisoners in Prison of Paços de Ferreira (EPPF) during the period from December 2021 to July 2022.

The study was submitted and approved by the ethics commission of the University Institute of Health Sciences, with reference CE/IUCS/CESPU-20/21 and by the General Directorate of Reintegration and Prison Services.

-Study Population and Data Collection

The present study was conducted the prisoners in Prison of Paços de Ferreira (EPPF) during the period from December 2021 to July 2022.

Patients were carefully informed through oral and written explanations about the purpose and procedures of the study. Patients who agreed to participate in the study were asked to sign an informed consent and to complete a questionnaire before the clinical examination. Inclusion criteria were patients’ presence at the EPPF during the data collection period and age of 18 years or older. Exclusion criteria were males subjects under 18 years old and individuals under disciplinary isolation. The subjects who did not give their consent were excluded.

The sample was selected according to a non-probability convenience sampling method from the inmate population of the EPPF. The study population in this cross-sectional clinical study comprised 107 male prisoners. Of the 107 prisoners, 4 were excluded from the study because they refused to participate. Details of the nature and purpose of the study were described verbally, and in writing, using the consent form. Written informed consent was obtained from all participants before undergoing oral examination and questionnaire.

The sample was subjected to a questionnaire and clinical examination. The following information was collected: sociodemographic data; level of education; eating habits; smoking habits and oral hygiene practices.

The variables collected through clinical examination were: number of decayed, missing and filled teeth; number of teeth with mobility, pocket depth (PD), measured as distance from the gingival free-margin from the bottom of the pocket; gingival recession (REC) as the distance from the enamel-cement junction (CEJ) to the free gingival margin; clinical attachment loss (CAL); Plaque Index (PI) and Bleeding on Probing (BoP).

-Dental status 

For the evaluation of dental caries, the DMFT index was used, considering the criteria defined by the World Health Organization (WHO) (10). Thus, teeth were considered healthy: when no clinically identifiable caries lesion was found, with or without treatment; even if white or discolored spots were present, provided there was no cavitation or softened tissue on probing. Teeth with evidence of trauma were excluded from this group, as it was not possible to ensure the absence of an endodontic lesion. Decayed teeth were defined as teeth with clinically visible cavitation or sulci with softened tissue on probing. Also included in this group were teeth with provisional fillings, or teeth filled with caries, regardless of being a primary lesion or relapse. Elements that were not suiTable for treatment due to coronary destruction were excluded from this group and registered as teeth with extraction indication. A missing tooth was defined as: when there was absence of dental elements, either due to caries, when it was possible to ensure that the loss was due to dental caries, or for another reason other than caries, when it was not possible to ensure that it was due to caries. In this group were included the elements registered with indication of extraction. A tooth was considered as filled: when it presented one or more restorations, without any visible sign of caries. Fixed dental prosthesis/bridge abutment, special crown, or veneer/implant) are not included in calculations of the DMFT index.

-Periodontal status 

Periodontal health, gingivitis and periodontitis were defined according to the new consensus of the AAP/EFP (11). Periodontal Health was when the total percentage of bleeding in probe was <10% and probing depth ≤ 3mm. Gingivitis was when the total percentage of bleeding in probe was ≥ 10%. Periodontitis was considerable when interproximal CAL was detecTable in two or more interproximal sites not adjacent or there was an interproximal CAL of 3 mm or more, non-vestibular or lingual/palatal, for ≥ 2 teeth. The periodontitis stage was defined according to the severity of the extension. For staging, interdental CALs of loss of 1-2mm, 3-4 and ≥5mm were considered mild (stage I), moderate (stage II) and severe (stage III) very severe (stage IV), respectively. The presence of complex factors of stage modifiers implies that the stage is altered for a higher stage. Stage IV was differentiated from stage III by the modifying factors including greater than or equal to 5 teeth lost due to periodontal injury; presence of masticatory dysfunction; secondary occlusal trauma; severe alveolar bone defect, < 20 remaining teeth. For progression, presence of % bone loss per year < 0.25 mm, 0.25-1 mm and >1 mm was considered as slow progression (grade A), moderate progression (grade B) and rapid progression (grade C), respectively. The presence of risk factors implies that the grade is altered. Grade C was differentiated from B by risk factors which are: smoker of ≥ 10 cigarettes per day and HbA1c ≥ 7.0% patient with diabetes. Extension of periodontal disease was classified as localized (<30% of teeth involved) or generalized (> 30% of teeth involved).

-Statistical Analysis

The Data were analyzed using IBM SPSS (Statistical Program for Social Sciences), version 28.0 for Windows. Descriptive statistics were expressed as mean, standard deviation for quantitative variables and as frequencies and percentages for qualitative variables. The data were initially examined for normality with the Shapiro-Wilk test and, if they did not reach normality, the analyses were performed by non-parametric methods. In this sense, we alternated between parametric and non-parametric analysis. To compare the DMFT according to the consumption or not of illicit substances, we used the independent t-test. Cohen’s d was used to calculate the effect sizes. The following guidelines were observed: | d |≤ 0.20 interpreted as a small emotional fear effect size, | d |= 0.50 as a moderate emotional fear effect size, and | d |≥ 0.80 as a large emotional fear effect size.

To compare the periodontal indexes between the different groups the Kruskal-Wallis test was used, followed by the Dunn test with Bonferroni correction. To buy the periodontal indexes between smokers and non-smokers, the Mann-Whitney test was used. The Chi-square test was used to assess the association between qualitative variables, age groups and smoking habits, according to periodontal condition. The level of statistical significance used was ≤ 0.05.

## Results

-Demographic and Socioeconomic Data

Our sample was composed of 103 inmates aged between 25 and 75 years (mean = 41.58; standard deviation = 8.94), most of whom were aged between 36 and 44 years (39.8%) (Table 1). As regarding education, 30 (29.1%) of the participants had attended the 2nd cycle, 29 (28.2%) had attended the 3rd cycle and 24 (23.3%) had attended the primary school. Only 3 (2.9%) of the participants had completed higher education and 17 (16.5%) had completed secondary education (Table 1).

**Table 1:**
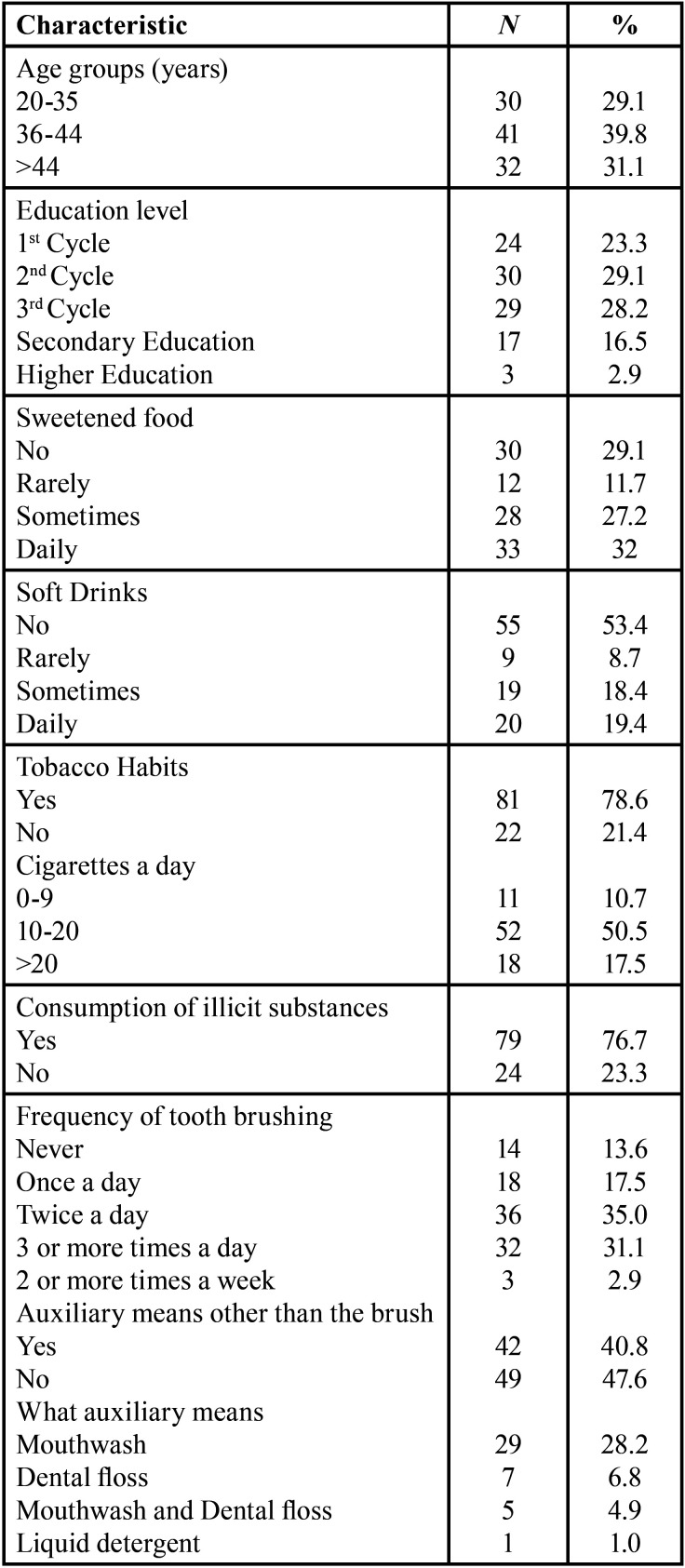
Selected characteristics of the study population.

-Habits: Dietary, smoking, and oral health

Most of the participants (70.9%) said that they ingest sugary foods, and 32% of them admit consuming those daily. Forty-five-point six percent (45.6%) of the participants reported drinking soft drinks and 19.4% do it on a daily (Table 1). Most of them are smokers (78.6%) and 52 (50.5%) smoke between 10 and 20 cigarettes a day. Only 22 (21.4%) individuals were non-smokers. Regarding the consumption of illicit substances, it was found in 76.7% of the participants, and only 23.3% did not consume (Table 1).

From the analysis of Table 1, regarding the oral hygiene habits, 89 (86.4%) of the participants mentioned brushing their teeth and of these 36 (35%) say they do it twice a day and 42 (40.8%) say they use, besides the brush and paste, mouthwash and/or dental floss as an auxiliary means of brushing.

-Dental caries and Risk Factors

The mean DMFT of the sample was 17.17 ± 8.23. The most prominent components were the number of lost teeth, with mean value of 13.14 ± 8.32, followed by the number of filled teeth, with mean value of 2.17 ± 2.17. The least relevant component was the number of decayed teeth, with a mean value of 1.86 ± 2.48.

It was observed that 7 (6.8%) subjects are totally edentulous, 64 (62.1%) have caries lesions and 32 (31.1) have no caries lesions.

When we analyzed the prevalence of caries lesions according to age group, it was found that of the 64 individuals who presented caries lesions, the highest prevalence was in individuals aged between 36 and 44 years (40.6%), but this relationship did not statistical significance (Table 2).

**Table 2:**
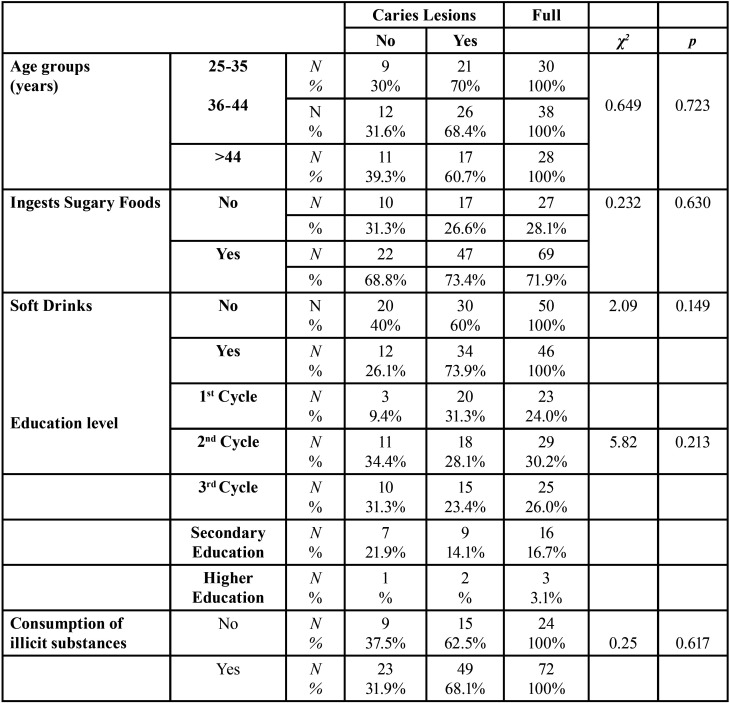
Caries lesion and age, intake of sugary foods and soft drinks and education level.

In Table 2, we can see that of the 64 individuals presenting caries lesions, 47 (73.4%) eat sugary foods and 17 (26.6%) do not eat sugary foods, however this relationship is not statistically significant.

When we analyzed the relationship between soft drink intake and caries lesion, it was found that of the 46 individuals consuming soft drinks, the majority (73.9%) had caries lesion, however this relationship is not statistically significant (Table 2).

Regarding the relationship between caries lesions and the consumption of illicit substances, it was found that this did not reach statistical significance, however, of the 72 individuals who consumed illicit substances, the great majority (68.1%) had caries lesions and 31.9% did not (Table 2).

When we relate caries lesion with education (Table 2), we found that more than 50% of the 64 individuals with caries lesion have completed the 1st or 2nd cycle. Of the 23 individuals with 1st cycle, 20 (87%) presented caries lesions, however, this relationship is not statistically significant.

When we compared the mean DMFT values according to the consumption of illicit substances, it was found that users of illicit substances have significantly higher mean DMFT values (12.08 ± 6.56), compared to participants who do not consume drugs (18.95 ±8.39) (t(101) = -3.68; *p* < 0.001).

-Periodontal Diseases and Risk Factors

Analyzing the prevalence of periodontal diseases, it was found that out of the 96 inmates who have some teeth, 40 (41.7%) have periodontitis, 31 (32.3%) gingivitis and 25 (26%) periodontal health. Regarding the extent of periodontitis, in 54.8% of the inmates it was localized and in 52.5% it was generalized. Of the 31 of inmates with gingivitis, 54.8% had a localized extension and 45.2% a generalized extension.

As regards the severity of periodontitis, the most frequent stage was I-Initial (57.5%), followed by stage II-Moderate (27.5%). Only 2.5% presented stage IV-Very Severe and 12.5% stage III-Severe. As regards progression, the most frequent grade is grade C - Rapid Progression (92.5%). Only 2.5% shows grade A - Slow Progression. As regards the plaque index, the average was 43.95% and the BoP was 12.05%.

The Plaque Index was evaluated according to the periodontal condition. The comparison between the 3 groups, presented in Table 3, showed a significantly lower level of plaque index in the periodontally healthy subjects compared to the subjects with gingivitis (*p* = 0.026) and the subjects with periodontitis (*p* <0.001).

**Table 3:**
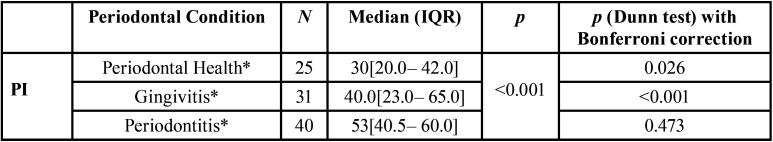
Comparison of the Plaque Index (PI) according to periodontal condition (Kruskall Wallis test).

In Table 4, there is a statistically significant relationship between periodontal condition and smoking habits (χ2 = 6.50; *p* = 0.039), with 36 (90%) of the 40 individuals with periodontitis being smokers.

**Table 4:**
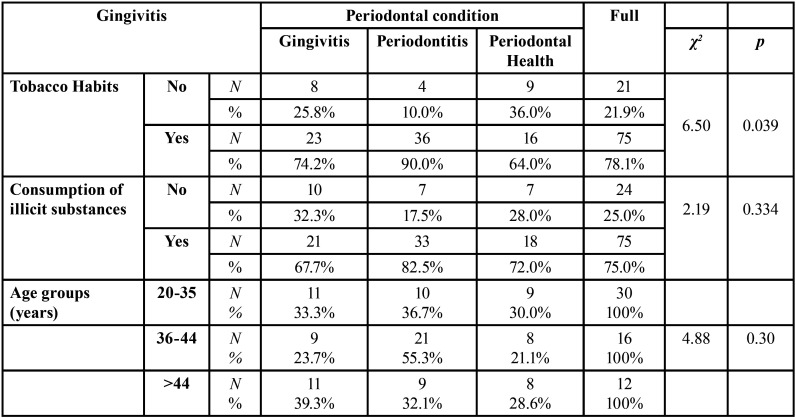
Periodontal condition according to smoking habits, consumption of illicit substances and age.

Regarding the consumption of illicit substances, 33 (82.5%) individuals with periodontitis, 21 (67.7%) with gingivitis and 18 (72.0%) with periodontal health were found to have consumed illicit substances, although this relationship is not statistically significant (Table 4).

Table 4 also shows that the highest prevalence of periodontitis occurs in the age group 36 to 44 years and that of the 40 individuals with periodontitis, 21 (55.3%) are in this age group. Despite these values, there is no statistically significant relationship between age and periodontal condition.

## Discussion

The promotion of health, and oral health, among the prison population is currently a necessity, but also a major challenge. In this context and given the lack of studies on oral health problems in Portugal, this study is an important contribution, providing new scientific evidence on the state of oral health in prison settings.

-Sociodemographic variables

The study population comprised one prison establishment, in the North of Portugal, and was composed of 103 individuals. Although small, the number of participants is in line with international studies, namely those carried out in the United Kingdom (n=78) (3), France (n=84) (12), Finland (n=100) (13) and Brazil (n=127) (14).

Analogously to the studies developed by Reddy *et al*., (2012) (15) and Nobile *et al*., (2007) (5), most of the participants in our study belong to the age range of 36-44 years, with a mean age of 41 years.

Regarding education, most of our sample, 57.3%, had attended basic education (2nd and 3rd cycle), and only 3 of the inmates had attended higher education. These results are corroborated by the studies of Leite Cavalcanti *et al*., (2014) (14) where they found that most participants had completed basic education (67.7%), and Vainionpää *et al*., (2017) (13) where they reported that 68% completed compulsory education. Other study, conducted in India (16) noted that majority of the respondents had no level of education.

-Health and oral health behaviours

A UK prison study (3) found that a high number of participants consumed sugary foods and drinks between meals. Similarly, a study conducted in Finland (13), found that almost two thirds of respondents consumed sugary foods daily. The same was observed in the present study, with most of the inmates (70.9%) opting for an unhealthy diet rich in sugar. People from lower social classes are more likely to demonstrate unhealthy behaviours (3).

The present study showed a high smoking prevalence (78.6%), which can be corroborated with other studies that observed a smoking prevalence of 70-80% (3). Values higher than 80% were found in the studies conducted by Priwe *et al*. (2018) (17) and Vainionpää *et al*. (2017) (13).

Inmates justify their unhealthy lifestyle, in particular sugar intake and smoking, to cope with the high levels of stress in prison (3).

With regard to oral hygiene habits, 86.4% of participants reported brushing their teeth and most participants had a frequency of brushing twice a day (35%). Values slightly lower than those found in other prison populations, with the exception of the study carried out in India, where 66.6% said they had brushing habits. Studies conducted in Italy (5), Australia (7), Nepal (16), Finland (13) found values higher than 90%. As observed in our sample, brushing frequency was daily in these studies (5,7,13,16).

The sample under study reported brushing their teeth daily, but the observed plaque index was moderately high (43.95%). The prisoners’ knowledge about good oral hygiene practices may be limited, namely the correct brushing technique, the use of brushing aids (dental floss and/or brush), and also the change of toothbrush every 3 months.

-Oral health and associated factors

Only 28 teeth were counted for the evaluation of the DMFT, excluding the third molars. The mean DMFT value in the present study was 17.17. Similarly high values between 10-22.5 were observed in China (18), UK (3), France (12), Brazil (14), Australia (7), Finland (13), Italy (5), Sweden (17) and Scotland (19). On the other hand, lower values were observed in India (15) and Kosovo (20).

The component with the highest weight was the number of teeth lost, with a mean value of 13.14, followed by the number of teeth filled, with a mean value of 2.17. Total tooth loss was verified in 7 inmates. The component with less relevance was the number of decayed teeth, with a mean value of 1.86. The demand for Dental Medicine services inmates may influence the lower number of decayed teeth and the higher number of teeth lost by treatment or extraction.

Walsh *et al*. (2008) (9) reviewed studies originating from Europe, South Africa, Australia, China and the USA and concluded that inmates have more decayed and lost teeth and DMFT equal to or greater than individuals from similar groups in the general population.

Cavalcanti *et al*. (2014) (14) observed that the component with the highest value was the number of decayed teeth, with a mean value of 11.06. This difference may be associated with cultural issues, namely the type of diet, as well as the adoption of different methodologies by the researcher. The use of radiographs allows the identification of interproximal caries, which may not be detected in clinical examination, thus contributing to the increased detection of caries lesions.

Regarding the prevalence of diagnosed oral diseases, the results of the present study indicated that caries lesion was the most prevalent (62.10%). When compared to other study that reported a prevalence of 98%, this result is relatively low. It may be due to differences in dietary and oral hygiene habits and or due to lack of provision of dental services (5).

Nagarale *et al*. (2014) (21) report that 98% of inmates never received any dental treatment in prison.

The combination of unhealthy eating habits and sugar intake presents an association with the appearance of caries lesion. The frequent consumption of sugary food is associated with a greater development of caries due to the greater frequency of demineralization cycles of the dental structure (22). This association was found in the present study without, however, reaching statistical significance. Of the 64 individuals with caries lesions, most (73.4%) eat sugary foods and only 17 inmates do not eat sugary foods. It is known that sugar consumption is likely to be a more relevant indicator for the risk of developing dental caries in individuals who do not have regular exposure to fluoride.

The study in Iran of oral health and its determinants among opiate dependents revealed poor oral health in terms of dentition and periodontal health. Missing teeth comprised the main part of their dental caries history and none of them had a healthy periodontium. Shekarchizadeh *et al*. (2019) observed regarding periodontal health, older dependents and those who started drug abuse at a younger age were more likely to develop periodontal pockets (23).

Regarding the prevalence of periodontal diseases, periodontitis was the most prevalent periodontal disease observed in our sample (41.7%), mainly in the age group between 36 and 44 years. The results of this study, regarding severity, indicated that mild periodontitis was the most prevalent. As for progression, rapid progression is the most frequent (92.5%) and with regard to extension, a higher prevalence of localized extension was observed. According to international studies conducted in Nigeria (24) and Russia (25), results in the range of 35-56%, were reported for prevalence of periodontitis. Balkrishna *et al*. observed that 64.20% had moderate periodontitis (26).

A prevalence of periodontal health was observed at 26%, which is higher than study conducted in Sweden (17). This may be due to the lack of oral health services in the prison where these studies were conducted or the majority of the participants are under 33 years of age.

The present study showed a prevalence of gingivitis of 32.3%, a value very similar to that obtained in a study carried out in a prison population in Russia, where a prevalence of gingivitis of 33.1% was observed (25).

Regarding PI, the mean value observed in the present study was 43.95%. Similar results was obtained in the study by Dayakar *et al*. (2014) (6).

In this study there were statistically significant differences in PI according to periodontal condition. Individuals with periodontal health had lower median PI values (median = 30.0) compared to individuals with gingivitis (median = 40.0) and periodontitis (median =53). The level of education is associated with an increased risk for the development of periodontal diseases, namely periodontitis (22). Bhattarai *et al*. report that participants with low level of education had higher prevalence of gingivitis and periodontitis (27).

 It was possible to observe a higher prevalence of periodontitis in individuals who attended primary schools. The fact that these individuals show less concern with oral health is one of the possible explanations.

It was found that periodontitis is clearly higher in smokers, the relationship between the two was statistically significant. Our results are in agreement with other studies, which found a relationship between smokers and the presence of periodontal pockets (28).

This study had some limitations. We observed difficulties for obtain a representative sample, since the sample was relatively small. We could not use sample power calculation as this was a convenience sample and limited by the existing individuals that could participate in the study after applying our inclusion/exclusion criteria. Although we cannot exclude the existence of some bias or some lack of statistical associations in our study, this is one of the few existing data on Portugal regarding this sample characteristics, which contribute for the increase in the knowledge of oral health of these populations. Another limitation found was the fact that the questionnaire was completed in the presence of the researcher, and the respondents may have felt some pressure to answer in a more socially accepted way.

## Conclusions

The analysis of DMFT in this sample was relatively high with the number of lost teeth with the greatest weight and the number of decayed teeth with the lowest expression. According to the current classification regarding severity, progression and extension, the most prevalent periodontitis was localized Stage I, Grade C Periodontitis. The prevalence of oral diseases diagnosed in this prison population is high, as well as the loss of dental pieces. Caries lesion is the most prevalent oral disease, and regarding periodontal diseases, it is periodontitis. Regarding the potential risk indicators of caries lesion a trend was observed between the combination of unhealthy eating habits and sugar intake with the appearance of caries lesion. Plaque index and smoking habits proved to be potential risk indicators of periodontal diseases.
